# Anticancer drug R&D landscape in China

**DOI:** 10.1186/s13045-020-00877-3

**Published:** 2020-05-13

**Authors:** Shen Zhao, Hongyun Zhao, Cheng Lv, Jifang Gong, Jian Zhang, Wenfeng Fang, Jin Li, Xichun Hu, Yi Ba, Binghe Xu, Yanqiao Zhang, Yun Fan, Kunyan Li, Xiaoyuan Chen, Zhimin Yang, Lin Shen, Li Zhang

**Affiliations:** 1 Department of Medical Oncology, State Key Laboratory of Oncology in South China, Collaborative Innovation Center for Cancer Medicine, Sun Yat-sen University Cancer Center, Zhongshan School of Medicine, Sun Yat-Sen University, 651 Dongfeng East Road, Guangzhou, 510060 China; 2 Department of Clinical Research, State Key Laboratory of Oncology in South China, Collaborative Innovation Center for Cancer Medicine, Sun Yat-sen University Cancer Center, Zhongshan School of Medicine, Sun Yat-Sen University, Guangzhou, China; 3 Department of Translational Medicine, CSPC Pharmaceutical Group Limited, Shanghai, China; 4 Department of Gastrointestinal Oncology, Key Laboratory of Carcinogenesis and Translational Research (Ministry of Education), Peking University Cancer Hospital and Institute, 52 Fucheng Road, Haidian, Beijing, 100142 China; 5 Department of Medical Oncology, Fudan University Shanghai Cancer Center, Shanghai, China; 6 Department of Oncology, Shanghai East Hospital, Tongji University School of Medicine, Shanghai, China; 7 Department of Medical Oncology, Tianjin Medical University Cancer Institute and Hospital, National Clinical Research Center for Cancer, Key Laboratory of Cancer Prevention and Therapy, Tianjin’s Clinical Research Center for Cancer, Tianjin, China; 8 Department of Medical Oncology, Cancer Hospital, Chinese Academy of Medical Sciences & Peking Union Medical College, Beijing, China; 9 Department of Gastrointestinal Oncology, Harbin Medical University Affiliated Cancer Hospital, Harbin, China; 10 Department of Medical Oncology, Zhejiang Cancer Hospital, Cancer Hospital of University of Chinese Academy of Sciences, Hangzhou, China; 11 Department of Medical Oncology, Hunan Cancer Hospital, The Affiliated Cancer Hospital of Xiangya School of Medicine, Changsha, China; 12 National Center for Drug Evaluation, National Medical Products Administration, Beijing, China

China’s drug regulatory reform since 2017 is significantly reshaping its drug R&D ecosystem and biopharmaceutical industry. The Chinese Phase 1 Oncology Trial Consortium, a collaborative group dedicated to early-phase clinical studies in oncology, conducted a comprehensive survey of China’s anticancer drug R&D landscape in its 2017 annual report [1]. In 2018, the Consortium has conducted another survey and compare the two to provide a longitudinal analysis of the changing landscape of early phase oncology trials in China and to shed some light on future strategies in anticancer drug R&D.

## Dramatic growth in phase 1 oncology trials

**Fig. 1:**
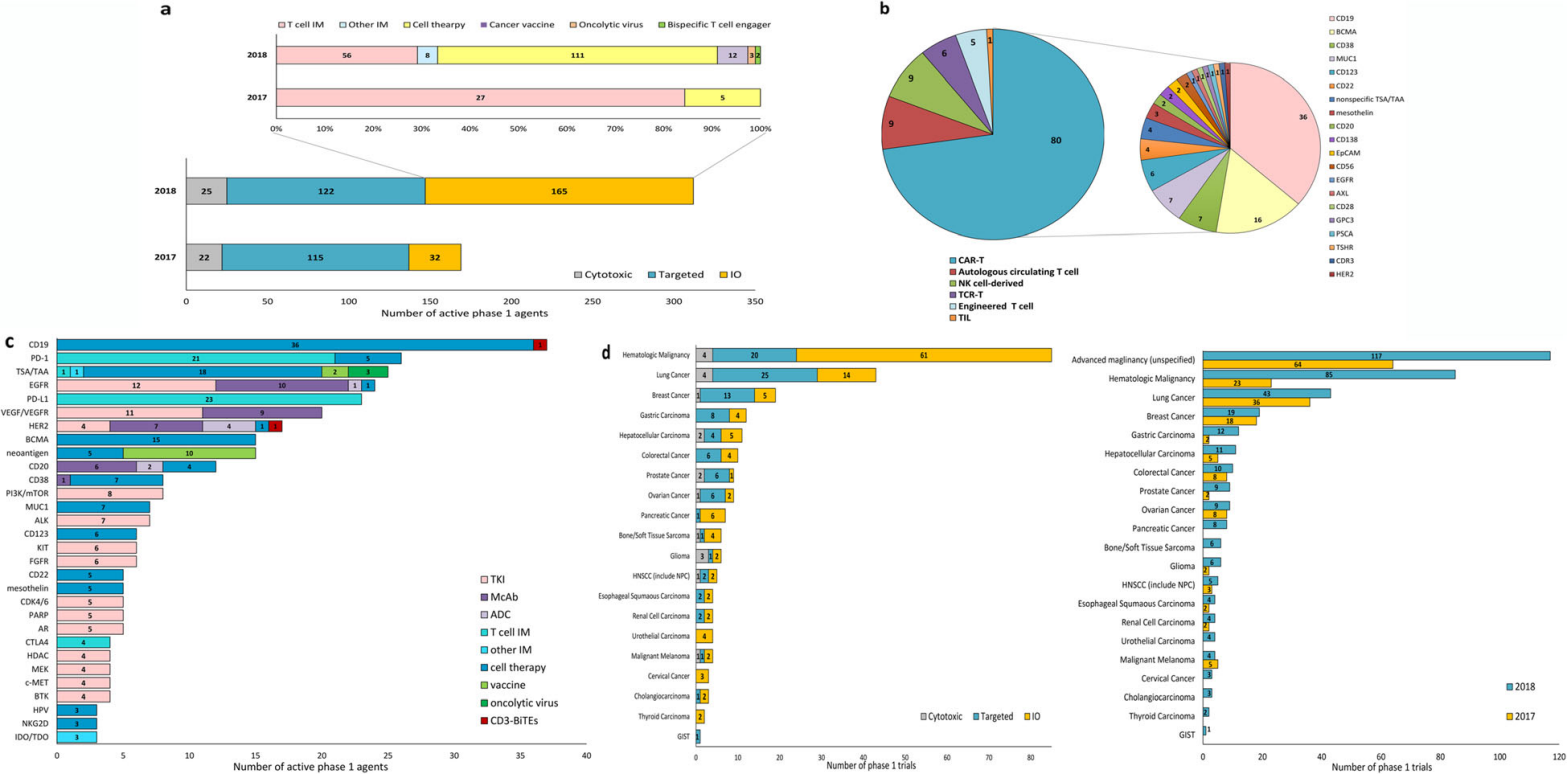
Phase 1 oncology pipeline, targets, and studied cancer types. **a** Overall phase 1 pipeline. **b** Cell therapy pipeline. **c** Top 30 targets in the phase 1 pipeline. **d** Most studied cancer types in phase 1 trials and changes relative to 2017

One year after the regulatory reform, there was a 102% increase in the number of phase 1 trials and 85% increase in the number of phase 1 agents in mainland China (Fig. 1a). A total of 312 agents were being tested in 364 phase 1 studies in 2018, tripling the requirement of phase 1 patient volunteers in a single year (7133 vs. 20,212). The number of first-in-human (FIH) studies also increased from 9% (16/180) to 15% (53/364) in the past year. In terms of treatment strategies, IO therapy has surpassed targeted therapy as the most popular strategy in anticancer drug R&D [1]. Fifty-three percent of the 312 agents belonged to IO therapies (*n* = 165), which was a 416% increase relative to 2017. Their action of mechanisms also becomes more diverse (Fig. 1a).

## Boom in cancer cell therapies and bispecific antibodies

Cancer cell therapy and bispecific antibody are the fastest-growing sectors. The number of phase 1 cell therapies increased from 5 to 111 in a single year. Types of cell therapy expanded from one single class (CAR-T) to six classes including CAR-T, autologous circulating T cells, NK cell-derived therapies, TCR-T, engineered T cells, and tumor-infiltrating T cells (Fig. 1b) [2]. Bispecific antibody (BsAb) is another emerging field. There were 13 BsAbs in phase 1 stage in 2018, including five PD-1 based agents, four CD3-based agents, and four HER2-targeted agents.

## Overcrowded CD19 and PD-1/PD-L1 pipelines

Targets tested in phase 1 oncology trials have grown from 28 to 64 in the past year. CD19 surpassed PD-1 as the most popular target in phase 1 studies (Fig. 1c) [1]. There were 37 CD19-targeting therapies being evaluated, 97% of which were CD19 CAR-T therapies (*n* = 36). PD-1/PD-L1 remains one of the hottest targets. Despite the NMPA (National Medical Products Administration) approval of five anti-PD-1 monoclonal antibodies (McAb), the number of phase 1 anti-PD-1/PD-L1 agents still increased from 27 to 49 in 2018, 44 among which were McAb. Nevertheless, novel PD-1/PD-L1 targeting therapies also emerged this year. Front-runners in this pipeline have shifted their focus to BsAb or cell therapy. HengRui and Innovent Biologics, for example, are respectively testing their PD-L1/TGF-βRII BsAb (SHR1701) and PD-1/PD-L1 BsAb (IBI318) in phase 1 studies.

## More diverse cancer types studied in phase 1 trials

67.9% (247 trials) of phase 1 oncology trials enrolled patients with specific types of cancer. The top five most-studied cancers in 2018 were hematologic malignancy (85 trials), lung cancer (43 trials), breast cancer (19 trials), gastric carcinoma (12 trials), and hepatocellular carcinoma (11 trials) (Fig. 1d). Notably, hematologic malignancy had a 270% increase in the number of trials relative to 2017. More phase 1 studies targeted characteristic malignancies in China (gastric carcinoma, hepatocellular carcinoma, esophageal carcinoma, and nasopharyngeal carcinoma) (Fig. 1d). Malignancies that failed to respond to current treatments also started to gain more attention (e.g., pancreatic cancer) (Fig. 1d).

## Expansion of phase 1 study sites and study scale

**Fig. 2:**
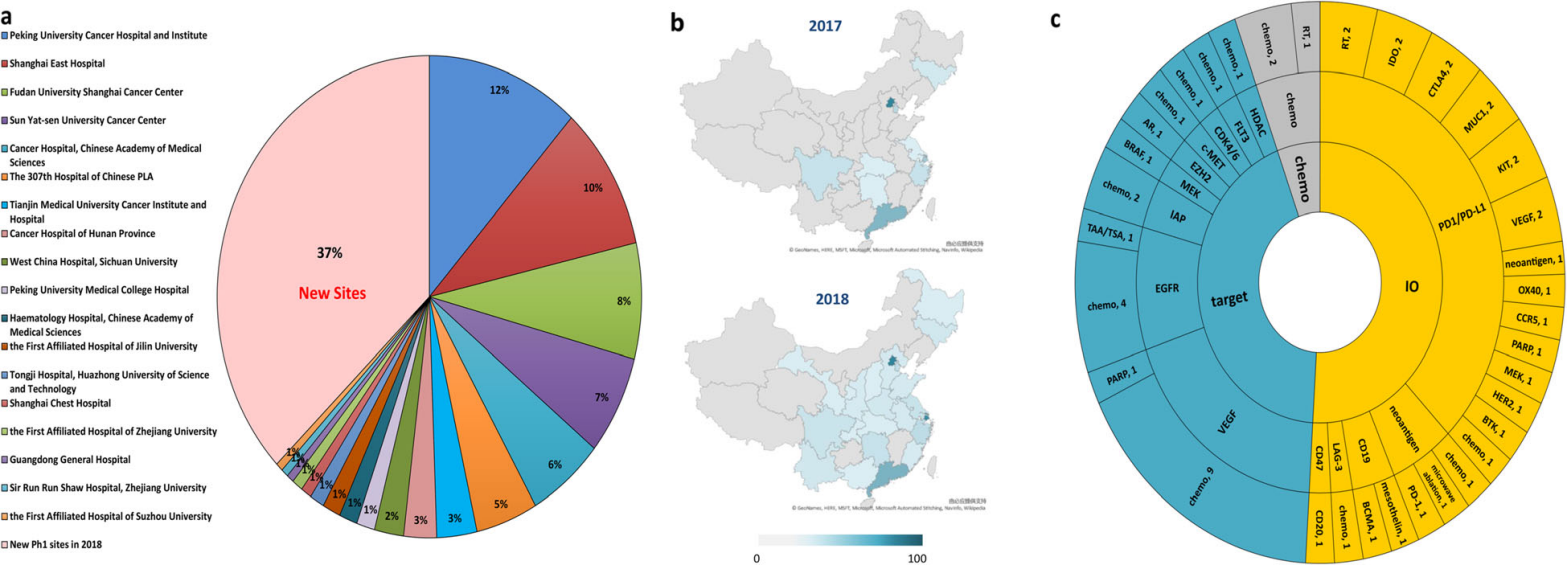
Phase 1 study sites and strategies in combination trials. **a** Three hundred sixty-four phase 1 studies were took on by 83 phase 1 study sites. **b** Geographic distribution of phase 1 study sites. **c** Combination strategies in phase 1 trials

Consistent with the growth in phase 1 trials, phase 1 study sites also increased significantly and showed a more balanced geographic distribution (Fig. 2a, b). Three hundred sixty-four phase 1 studies were took on by 83 phase 1 study sites at 22 different provinces across China. Although the majority of phase 1 trials were still conducted by faculties in Beijing, Shanghai, and Guangzhou, the proportion has dropped from 73% (236/364) to 65% (131/180). Furthermore, the scale of phase 1 oncology trials has evolved from small single-site studies to oligo-site (2 to 4 participating centers, *n* = 32) or multi-institutional studies (≥ 5 participating centers, *n* = 8). The average number of patients required for each trial increased from 40 patients to 56 patients per trial.

## Investigator-initiated trials played a greater role in early-phase studies

In terms of sponsorship, 71% of the phase 1 trials (*n* = 258) were sponsored by domestic biopharmas, 3% (*n* = 10) by multinational corporations (MNCs), and 26% (*n* = 96) were investigator-initiated trials (IITs). The percentage of MNC-sponsored trials has further dropped in the past year [1]. No MNC-sponsored global phase 1 trials was conducted in China. Meanwhile, there was an interesting increase in the number of IITs (5 vs. 96), which may indicate a more permissive attitude towards early-stage IITs in the era of IO. Phase 1 IITs tended to be smaller in size and more exploratory in design. Their average enrollment target was 33 patients. Forty-seven trials (49%) included biomarker assessment in exploratory objectives, and 33 (34%) contained multiple (≥ 3) expansion cohorts.

## Increasing emergence of novel-novel combination trials

14.3% of the phase 1 oncology studies were combination trials (*n* = 52), which investigated 34 combination strategies covering 17 kinds of malignancies. IO therapies were the most studied combination strategies (Fig. 2c). Fifty percent of these trials (*n* = 26) contained at least one IO agents, 77% (*n* = 20) of which were anti-PD-1/PD-L1 McAb. We also noticed that 25% of these combination trials (13/52) involved two or more novel agents (novel-novel combination trial). This percentage is higher than the 9% we reported in 2017 and also higher than the 3% (49/1105) reported in the global analysis of IO trials [1, 3].

## Concluding remarks

After the drug regulatory reform, phase 1 oncology trials in China had experienced significant growth in multiple aspects. Anticancer drug R&D in China are paying more attention to its characteristic malignancies and diseases with unmet medical needs. FIH studies and exploratory IITs also increased considerably. However, remained gaps after the reform include the lack of originally designed agents, the absence of global phase 1 studies, and the need of more comprehensive regulations over novel-novel combination trials.

## Data Availability

All data generated or analyzed during this study are included in this published article and its supplementary information files.

## Abbreviations

R&D: Research and development
NMPA: National Medical Products Administration
IO: Immuno-oncology
CAR-T: Chimeric antigen receptor T cell immunotherapy
TCR-T: T cell receptor T cell immunotherapy
TIL: Tumor-infiltrating T cells
BsAb: Bispecific antibody
TSA: T cell-specific antigen
TAA: T cell-associated antigen
IIT: Investigator-initiated trial
PD-1: Programmed cell death-1
PD-L1: Programmed cell death-ligand 1
TGF-βRII: Transforming growth factor β Receptor II
IND: Investigated new drug
BCMA: B cell maturation antigen
